# Managing the challenges in implementing digital mental health in europe

**DOI:** 10.1192/j.eurpsy.2021.58

**Published:** 2021-08-13

**Authors:** O. Vlijter

**Affiliations:** Bod, ARQ National Psychotrauma Centre, Diemen, Netherlands

## Abstract

**Abstract Body:**

The demand for mental health care is increasing globally as a result of societal challenges such as automation, increased economic competition, unemployment and the growing impact of climate change. The direct and indirect economic costs of mental health problems are substantial, totalling over € 600 billion yearly across the EU (OECD 2018). The COVID-19 crisis has led to an additional increase in demand and has changed the way care is delivered. Since March 2020 there has been a significant increase in the use of e-mental health (eMH), telemental health in particular. eMH can contribute to keeping services, accessible, affordable and patient focused. The eMEN project (funded by the EU Interreg North-West-Europe programme) is promoting the latter through a European cooperation platform for eMH development, research and implementation. This platform focuses on high quality and professional ‘blended care’, which combines face-to-face and online treatment. The implementation of eMH has been slow and varies considerably between EU countries, even though this technology has been on the market for over 20 years. The reasons for this are related to quality problems (e.g. validation, usability), resistance from clinicians, lack of blended care treatment protocols, digital skills, reimbursement systems and policies and other barriers. Many service providers and public health authorities are increasing their efforts to overcome these barriers. This presentation will give a short overview of how the eMEN project is trying to overcome these barriers and accelerate the eMH implementation process.

**Disclosure:**

No significant relationships.

